# Degree Adjusted Large-Scale Network Analysis Reveals Novel Putative Metabolic Disease Genes

**DOI:** 10.3390/biology10020107

**Published:** 2021-02-03

**Authors:** Apurva Badkas, Thanh-Phuong Nguyen, Laura Caberlotto, Jochen G. Schneider, Sébastien De Landtsheer, Thomas Sauter

**Affiliations:** 1 Systems Biology Group, Department of Life Sciences and Medicine, University of Luxembourg, L-4365 Esch-sur-Alzette, Luxembourg; apurva.badkas@uni.lu (A.B.); sebdelandtsheer@gmail.com (S.D.L.); 2 Megeno S.A., L-4362 Esch-sur-Alzette, Luxembourg; phuong.nguyen@megeno.com; 3 Aptuit Center for Drug Discovery and Development, 37135 Verona, Italy; Laura.Caberlotto@aptuit.com; 4 Department of Internal Medicine II, Saarland University Medical Center, D-66424 Homburg, Germany; jochen.schneider@uni.lu; 5 Luxembourg Centre for Systems Biomedicine, University of Luxembourg, L-4365 Esch-sur-Alzette, Luxembourg

**Keywords:** metabolic diseases, co-morbidities, metabolic disease genes, networks, topology

## Abstract

**Simple Summary:**

To explore some of the low-degree but topologically important nodes in the Metabolic disease (MD) network, we propose a background-corrected betweenness centrality (BC) and identify 16 novel candidates likely to play a role in MD. MD specific protein–protein interaction networks (PPINs) were constructed using two known databasesHuman Protein Reference Database (HPRD) and BioGRID. The identified candidates have been found to play a role in diverse conditions including co-morbidities of MD, neurological and immune system-related conditions.

**Abstract:**

A large percentage of the global population is currently afflicted by metabolic diseases (MD), and the incidence is likely to double in the next decades. MD associated co-morbidities such as non-alcoholic fatty liver disease (NAFLD) and cardiomyopathy contribute significantly to impaired health. MD are complex, polygenic, with many genes involved in its aetiology. A popular approach to investigate genetic contributions to disease aetiology is biological network analysis. However, data dependence introduces a bias (noise, false positives, over-publication) in the outcome. While several approaches have been proposed to overcome these biases, many of them have constraints, including data integration issues, dependence on arbitrary parameters, database dependent outcomes, and computational complexity. Network topology is also a critical factor affecting the outcomes. Here, we propose a simple, parameter-free method, that takes into account database dependence and network topology, to identify central genes in the MD network. Among them, we infer novel candidates that have not yet been annotated as MD genes and show their relevance by highlighting their differential expression in public datasets and carefully examining the literature. The method contributes to uncovering connections in the MD mechanisms and highlights several candidates for in-depth study of their contribution to MD and its co-morbidities.

## 1. Introduction

Metabolism occurs in every cell of the body. It powers all the functions of the body, and disruption in the normal functioning of metabolism has systemic effects. Metabolic diseases (MD) consist of a cluster of disturbances—insulin resistance, hypertension, dyslipidemia, obesity, etc. [1,2], type 2 diabetes (T2D) [3] and are a risk factor for cardiovascular diseases (CVD) [4]—a leading cause of mortality. MD affect a large population currently and their incidence is projected to increase. It is a complex condition influenced by several factors such as genetics, diet, and environment [5]. MD are associated with several co-morbidities, such as non-alcoholic fatty liver disease (NAFLD), reproductive issues, and have been linked to cancer [6,7]. Co-occurrence of co-morbidities brings in the risk of increased medical complications, increased medicinal and health care costs, and has serious consequences on life expectancy. Hence, identifying genes particularly involved in such co-morbidities is of particular interest. While experimental data-driven methods such as genome-wide association studies (GWAS) have contributed to uncovering the genetic landscape of MD, these studies are expensive, require a large sample size, and often do not detect low-frequency mutations [8]. Neither do these allow for any mechanistic insights.

Computational analysis of networks offers another approach to understand the mechanism of diseases, highlighting potential candidates that could be prioritized for wet-lab validation. This work has been put forward as a promising way to study metabolic diseases from a system-level point of view [9,10,11,12,13,14,15,16,17]. The MD network has been explored in several previous studies. Among these, Li et al. investigated the relationships between human diseases and specific sub-groups of metabolic pathways to discover disease-metabolic sub-pathway [10]. Lee et al. and Goh et al. took advantage of network medicine to study both—the network of MD-related molecules, as well as the network of MD [9,15]. Recently, Lotta et al. carried out a two-stage meta-analysis to study metabolic health and then predicted its relevance to T2D [18]. Though previous works have successfully paved the way to the study of specific metabolic diseases, a comprehensive analysis of MD and their interactions remains challenging.

Several approaches for prioritization of gene candidates have been described in the literature to narrow down likely candidate genes. In general, these methods used the topology of protein–protein interaction networks (PPINs) together with various other data types to retrieve a measure of the importance of different genes or gene products in the regulation of the disease of interest [19]. Several methods are cancer-specific, often require quantitative patient data as inputs [20], while other methods require manual setting of parameters [21]. Data integration is a challenge since disparate sources have variations in gene names, data quality, etc. A recent review [19] has summarised the approaches and tools developed in the field.

To uncover novel disease-related genes, proximity to known disease genes is the basis for several methods [22]. One family of measures used to identify pivotal nodes in networks are centrality measures [23]. Betweenness centrality (BC) has been used to identify the nodes that are crucial for the flow of communication in the network [24], linking different parts of the network together. It is one of the most frequently applied measures in the literature [25]. Since networks have modules that tend to contribute towards a specific function [26] and nodes with high BC act as facilitators of interactions between such clusters, these central nodes could explain disease-associated co-morbidities. MD are particularly suited for analysis using this metric since several co-morbidities are seen, which, from a PPIN perspective, indicate nodes (proteins) that are likely to participate in multiple conditions, connecting different functional modules.

However, relying on centrality measures induces two types of bias. Firstly, biological data is noisy, incomplete, and includes false positives [27]. Secondly, some of the genes have been extensively studied, resulting in a literature bias towards them. The specific contribution of these heavily studied nodes to the disease in question needs to be determined. While the products of these genes play pivotal roles in many processes, their importance in specific mechanisms is often unclear. In other words, highly-connected genes will appear as important (high BC) for most subnetworks they are part of. The combination of these two biases attributes inflated importance to a small number of nodes and neglects low-degree, understudied genes that may be central to specific biological processes. The present study is interested in highlighting such nodes that may offer novel insights into disease mechanisms of co-morbid conditions of MD. Several methods have been proposed to address literature bias that results in degree dependence in such analyses. Erten et al. [21] propose several statistical approaches for addressing this bias. While this may increase the reliability of the outcomes, the method requires several inputs, and its applicability and usability may be restricted. Some of the prioritization methods integrate a host of data and require only gene-seed list input, such as ToppGene [28]. This method is restricted, for the gene prioritization method, to three options, and has limited applicability to investigate other topological metrics. While Biran et.al [29] propose a similar method as does this work, the reference networks generated by switching the edges from the original PPIN may not be biologically relevant.

The proposed method herein uses background-corrected BC as a measure, to analyze two PPINs. We used frequency of appearance in random networks, as well as the difference between centralities in MD and random networks as a two-pronged approach, identifying several significant genes that may be involved in MD associated co-morbidities. In this way, we ensure that the analysis draws attention to topologically crucial but lower-degree nodes involved in MD. Without this background correction, higher-degree nodes dominate the list of genes based on the raw BC centrality score. These are invariably genes that have been most frequently studied, and thus a literature bias has been introduced. The low-degree, topologically strategic genes identified would be good candidates for expanding the MD genes repertoire. We identified 16 novel genes shared by the two PPINs that show strong potential as MD genes. Pathway analysis and differential gene analysis of the identified significant genes highlights their pleiotropic roles in metabolic, immune, and central nervous systems.

## 2. Results

### 2.1. Identification of Novel Putative MD Genes 

**Figure 1:**
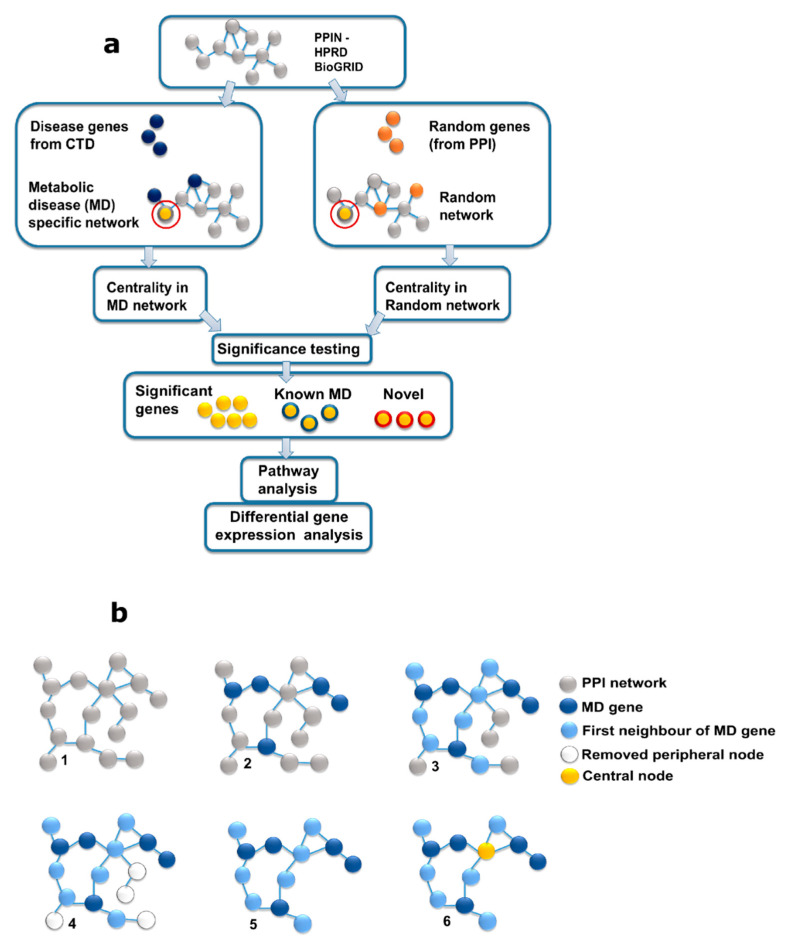
Graphical outline of the method; (**a**) outline of the proposed method; Comparative Toxicogenomics Database (CTD) and two protein–protein interaction (PPI) networks (Human Protein Reference Database (HPRD) and BioGRID) were used to build an metabolic diseases (MD)-specific network. To correct for degree bias, random networks were constructed using similar degree distribution as in the original MD network. On obtaining the centrality scores in the MD network and random networks, significance testing was carried out to assign *p*-values to the nodes. Nodes that showed significantly different centralities between the MD and random networks were subjected to a Pathway analysis. The novel genes identified were subjected to differential gene expression analysis. (**b**) MD specific network construction: 1. PPI giant component, 2. map-known MD genes onto PPI, 3.sselect first neighbors, 4. select interactions between first neighbors (if exist), 5. Remove single degree peripheral nodes (except MD), 6. centrality analysis of the network. Random networks for comparison were built using the same procedure, except, instead of the MD nodes from CTD, nodes were selected randomly from the PPI networks. The degree distribution of MD nodes in the MD-specific network was mimicked in the random networks.

**Figure 2:**
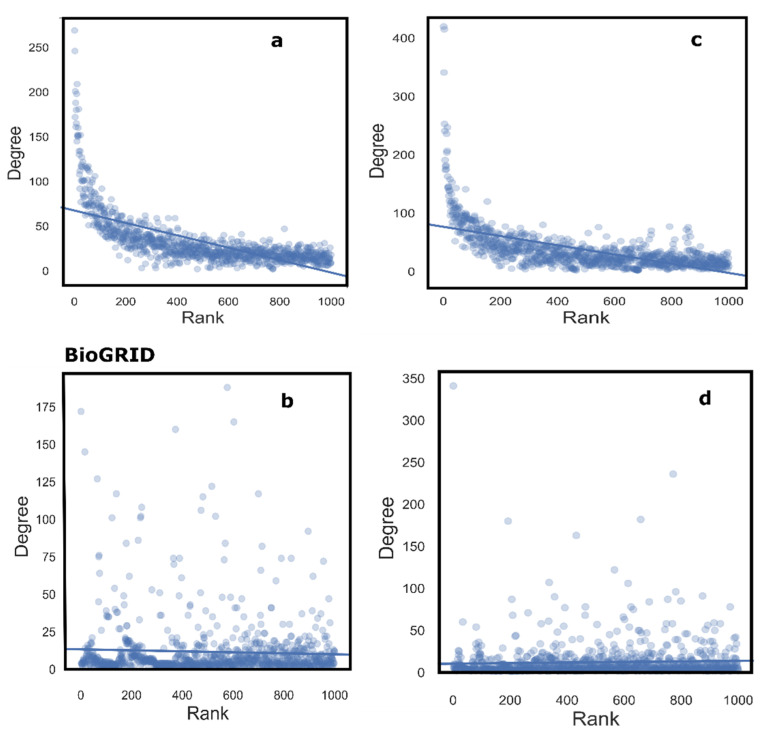
Correction of degree bias; Rank of top 1000 genes in HPRD (top) and BioGRID (bottom) based on (**a**,**b**): Betweenness centrality with no background correction and (**c**,**d**): *p* values. In (**a**) and (**b**), the highest-ranking nodes in the MD network are some of the highest degree nodes in the PPI network. In the background-corrected networks (**c**,**d**), we find a more uniform distribution of the ranking vis-à-vis the corresponding degree of the node. Some of the highly ranked nodes have degrees lower than 50. However, highly connected genes are also seen to be present in the top-ranking genes, which indicates that their contribution to the MD network is significant. Thus, the method allows for highlighting both low-degree and high-degree nodes in the top-ranking genes, which was not the case when only uncorrected betweenness centrality was used.

The increasing incidence of MD and its related co-morbidities have become a public health hazard worldwide. We developed a pipeline to identify genes that show a distinct contribution to the MD network and its co-morbidities, using BC as a metric to analyze the MD network (Figure 1), to correct for biases inherent in the data. We constructed an MD specific network, using the disease genes data from Comparative Toxicogenomics Database [30] (CTD), and mapping it to the two PPINs—Human Protein Reference Database [31] (HPRD), and BioGRID [32]. We chose to include four categories of metabolism-related disease areas—metabolic diseases, liver diseases, overnutrition, and undernutrition, bearing in mind the systemic nature of metabolism (Supplementary Table S1). The BC values in the MD network constructed using these seed genes were compared to those in degree-distribution adjusted and topologically comparable random networks. Statistical significance was assigned to each node based on this relative BC value. Degree bias is clearly seen in the correlation of the raw BC with the degree of the gene results (Figure 2a,b). While some of the high-degree genes may be involved in MD, their high connectivity indicates their interactions with different molecules, and thus, perhaps, diverse physiological roles. For their MD-specific contribution, we compared the centrality of the gene in the MD network to its centrality in random networks. Background-corrected BC-based ranking (Figure 2c,d) shows a more uniform degree distribution. A gene with a low *p*-value indicates a higher centrality in the MD network as compared to random networks. Thus, a gene with a low degree but strategically positioned in the MD network is likely to be identified by this approach (Supplementary Table S2). 

**Figure 3:**
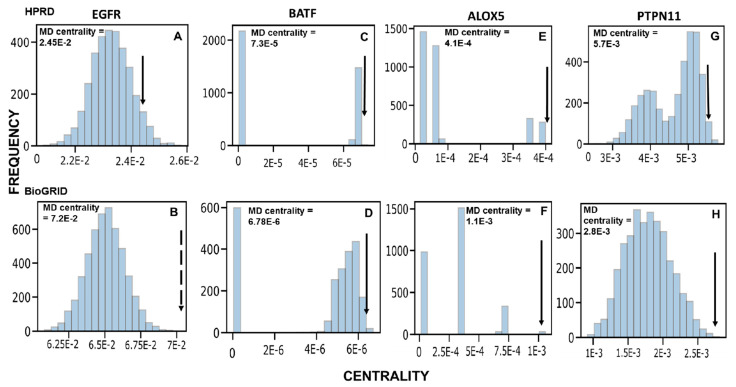
Centrality distributions of some genes in HPRD (top) and BioGRID (bottom); (**A**,**B**): EGFR; (**C**,**D**); BATF; (**E**,**F**): ALOX5; (**G**,**H**): PTPN11. MD centrality scores are the betweenness centrality values of these genes in the MD network. The black arrows indicate the MD centralities for the genes. All of the genes are significant in both the PPI networks (based on raw *p* values). For these genes, their centrality scores in random networks are rarely higher than their corresponding centrality in the MD network and hence have low *p* values.

To retrieve the most promising hits, we focused on the genes predicted by analyzing two different PPINs. Our method identified 602 and 288 genes with a corrected *p*-value < 0.05 for HPRD (Supplementary Table S3) and BioGRID (Supplementary Table S4), respectively, of which 39 genes were common. The genes found to be significant show a variety of centrality distributions (Figure 3), justifying the need to use non-parametric testing. These distinct distributions are most likely due to the scale-free nature of biological networks. In the case of highly connected genes such as EGFR, a variety of network configurations are possible, resulting in a normal distribution of centrality values. For smaller degree nodes, centrality values tend to be within specific intervals. 

Because our method is designed to retrieve those genes that have a discrepancy between their expected centrality and their MD-specific centrality, highly connected genes may be penalized. For example, TP53 is a known MD gene and has very high connectivity. However, its MD-specific centrality is comparable to its centrality in random networks (*p*-value 0.512 in HPRD, 0.407 in BioGRID), and thus assigned low priority by this method. This does not prevent high-degree nodes with high MD specific centrality from being highlighted, such as EGFR. EGFR is a highly connected gene in both PPINs, which also has significant MD-specific centrality in both (*p*-value: 0.0465 in HPRD, 0.0002 in BioGRID).

Out of the 602 significant genes in HPRD, 286 genes were part of the original seed list. Similarly, for BioGRID, 125 ones had been previously identified. Thus, 316 novel genes were identified in HPRD and 163 novel genes in BioGRID. The overlap yielded 16 novel candidates that are likely to be MD genes (Table 1). Some of the genes show extreme differences in their centrality values in the two networks. Prima facie, based on the number of interactions, the difference between HPRD (38,651 interactions) and BioGRID (42,666 interactions) is not notable. However, the two PPINs have only 11,047 interactions common between them. Therefore, given the low overlap, and thus, distinct interactions in the two networks, the overlapping 16 nodes—flagged as significant in both the networks—are likely to be robust candidates (See Supplementary Table S5 for details on PPIN size, hypergeometric test).

### 2.2. Pathway Analysis 

To examine the physiological context of the genes found to be significant in both the networks, the gene sets were analyzed to highlight the significant pathways these genes contributed towards. Pathway analysis for the two PPINs, performed using the two tools (Enrichr [33] and ConsensusPathDB [34]), shows convergence in some key pathways such as PI3K/Akt, JAK/STAT, and AGE-RAGE signaling pathway in diabetic complications. Some others of particular interest are the neurotrophic signaling pathway, FoxO signaling pathway. Despite the differences in the number of significant genes for the two PPINs, several of the pathways they were implicated in belong to the same broad category, such as hormone signalling, or pathways related to the immune system (Supplementary Table S6). The complete set of pathway analysis results can be found in the supplementary data (Supplementary Tables S7 and S8).

### 2.3. Differential Expression Analysis 

To examine the disease context/contribution of the 16 novel candidates, we looked at the differential expression of these candidates using Harmonizome [35]. This resource lists and provides links to, among others, the Gene Expression Omnibus (GEO) datasets that show, for the gene of interest, if it is found to be differentially expressed in different disease conditions. All of the 16 candidates were found to be differentially expressed across diverse conditions (Table 2, Supplementary Table S9). ALOX5 has been seen to be up-regulated in Down Syndrome, severe combined immunodeficiency (SCID), among others, and down-regulated in atherosclerosis. BATF is differentially expressed in MS (Multiple Sclerosis), diabetic nephropathy, cardiac hypertrophy, etc. PLSCR3 is seen to be associated with, among others, atherosclerosis, cardiomyopathy, myocardial Infarction, and bipolar disorder. Similarly, the other genes also showed differential expression in a host of other diseases. These genes are involved in diseases that can be grouped into three general categories: MD and co-morbidities, immune system conditions, and neurological disorders. For example, IL5RA is involved in cardiac failure, familial combined hyperlipidemia, and juvenile arthritis, and also Down Syndrome. While primary, familial hyperlipidemia is a hereditary condition, secondary hyperlipidemia has been linked to diabetes, among its causes. Arthritis is an autoimmune disorder. Down Syndrome is caused by chromosomal aberrations (inheriting an extra chromosome 21). Hampered neurological development along with other physical manifestations seen in such patients. The pathway analysis highlighting neurological developmental pathways, along with other neurodegenerative disease-related pathways, combined with changed expression of these genes in such conditions, indicate that these genes are involved in the functioning of neurodevelopmental/neurodegenerative conditions, along with the immune system and the metabolic system.

We believe these genes to be potential candidates for further study for their roles in MD and related co-morbidities.

## 3. Discussion

In the present study, we investigated to what extent the topological connectivity of MD associated gene networks is related to specific biological pathways and the co-occurrence of human MD. The genes with high BC likely play a crucial role in MD and its co-morbidities. MD has been linked to numerous co-morbidities such as cancers, psychiatric disturbances, psoriasis, auto-immune diseases such as lupus, mental disorders such as depression and schizophrenia, and several others [6,36,37,38,39]. Thus, a study of such genes is likely to yield better insights into the development and progress of MD related conditions.

Literature references for the 16 novel genes identified in this study were used to ascertain the validity of these genes as potential MD genes. Several of these genes are involved in multiple physiological processes, as envisioned by the use of BC. ALOX5 (Figures S1 and S2) shows a significant relative change in BC for both HPRD (Relative betweenness (MD centrality/Random network centrality) 3.95) and BioGRID (Relative betweenness 3.71). ALOX5 (Arachidonate 5-Lipoxygenase) encodes a member of the lipoxygenase gene family. ALOX5 is involved in the synthesis of leukotrienes from arachidonic acid, which are important immune mediators, and participate in several allergic and inflammatory responses. ALOX5 also plays a role in several cancers [40,41]. CTD associations of ALOX5 include asthma, atherosclerosis, insulin resistance, Alzheimer’s disease (AD), neurodegenerative diseases, dyslipidemias. Genetic Associations Database (GAD) associates ALOX5 with blood pressure, T2D, atherosclerosis, and AD. Gene Ontology (GO) biological processes associated include lipid metabolism and arachidonic acid metabolite production involved in an inflammatory response. Among ALOX5′s interacting partners, ALOX5AP, COTL1, and LCT4S have been identified as MD genes. Thus, ALOX5 has a strong potential as an MD candidate, and further investigation can be illuminating. 

**Figure 4:**
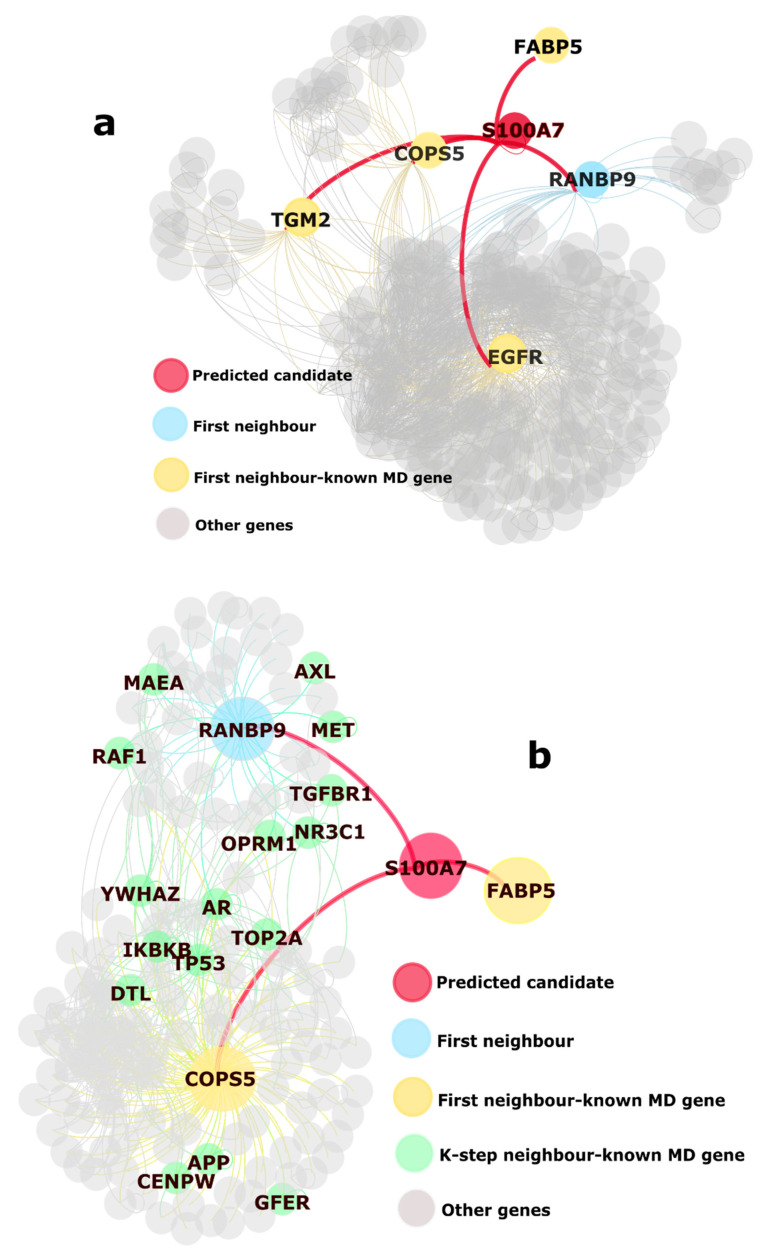
Example of significant gene S100A7 in: (**a**) HPRD and (**b**) BioGRID; In both the networks, S100A7 is connected to known MD genes. In HPRD, it connects some high-degree nodes such as EGFR. In BioGRID, it connects two clusters that have some important, known MD genes. The presence of APP here is notable since pathway analysis for these significant genes highlights Alzheimer’s disease. (Visualization: Gephi).

S100 Calcium Binding Protein A7 (S100A7, Figure 4), a member of the S100 family of proteins, has been found to be involved in various immune-system activities such as IL-17 signaling pathway, neutrophil degranulation, and chemotaxis [42]. This family plays a role in several processes, e.g., differentiation, cell cycle progression, cytoskeleton membrane interactions, intracellular calcium signaling, and cytoskeletal membrane interactions. Associated GO biological processes are immune response, response to stress and reactive oxygen species, regulation of cellular metabolites, and regulation of metabolism. CTD associates S100A7 with psoriasis, drug-induced liver injury, nervous system malformation, inflammation, and congenital heart defects. Increased expression of this gene, in the context of cancer, is associated with angiogenesis, increased tumor growth, and an increase in metastasis. Its interacting partners, FABP5, COPS5, and TGM2 are known MD genes.

Some of the common pathways highlighted by significant genes from the two PPINs show that these genes are involved in several important pathways crucial to metabolism. Dysregulation of the PI3K/Akt pathway is implicated in several diseases and accumulating evidence indicates that deregulation of the phosphatidylinositol 3-kinase (PI3K)/AKT pathway in hepatocytes is a common molecular event associated with metabolic dysfunctions including obesity, MD, and the non-alcoholic fatty liver disease (NAFLD) [43]. Our study also points to the role of inflammation and immune response in metabolic disorders, with the involvement of several interleukin and cytokine signaling pathways. Immune response and regulation of metabolism are highly integrated processes; dysfunction of which can lead to a cluster of chronic metabolic disorders [44]. Several studies point to the activation of the immune system due to a low-grade inflammation as a player in the pathogenesis of obesity-related insulin resistance and T2D [44,45,46,47]. An innate immune response can be activated during the development of the disease by dietary factors and endogenous damage-associated signals [48,49]. 

The FOXO family of transcription factors(TFs) have been linked to aging, cancer, and neurological diseases [50]. Some of the first identified targets of FOXO were metabolism and stress-resistance genes. FOXO is phosphorylated due to the activation of the PI3K-AKT pathway, due to the presence of insulin and insulin-like growth factor (IGF). These inhibit its activity, while conversely, in the absence of these factors, FOXO play their role of transcription.

**Figure 5:**
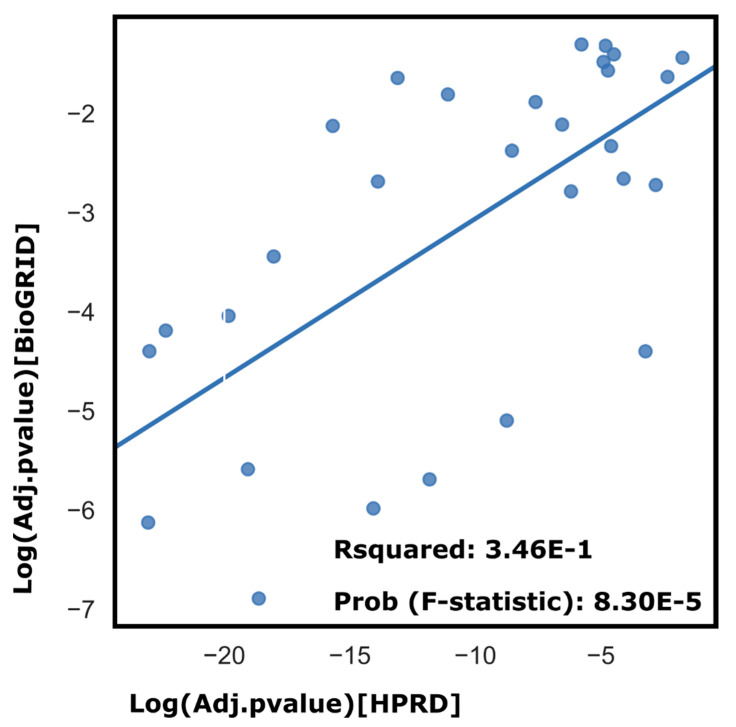
Correlation of pathways as highlighted by HPRD vs. BioGRID. Although the comparison here is for a different number of significant genes for the two datasets (602 for HPRD, 288 for BioGRID), resulting in different orders of magnitudes for the associated *p* values, the strength of the correlation is high.

Several pathways associated with neurodegenerative disorders (AD, Parkinson’s disease, and Huntington’s) were highlighted by the two different gene-sets from the two PPINs. Of particular interest is the AD pathway. For HPRD, the pathway is highlighted due to the presence of the genes APP, APAF1, LRP1, ITPR2, ATP2A2, ITPR3, ERN1, ATP5F1B, IL1B, UQCRC1, MAPK1, NDUFV2, and MAPK3, while for BioGRID, the presence of ATP5F1A, NDUFS6, COX4I1, NDUFS5, UQCRC2, APOE, PLCB1, ATP5F1C are responsible for highlighting Alzheimer’s. It is worth noting that the two different datasets yield different genes, but converge onto the same significant pathway. There is a significant correlation between the pathway analysis results yielded by the two PPINs (Figure 5). Research efforts have shown that factors like dyslipidemia, hyperglycemia, hypertension and obesity are parameters of the metabolic syndrome, but are at the same time, also risk factors for cognitive decline, i.e., represent a risk constellation for AD. Both AD and T2D share certain signs of dysfunctional mitochondria, which may lead to increased oxidative stress in the cells [51]. Insulin signaling has shown to be involved in protein tau processing, and human amylin, a beta cell peptide, has similarities to amyloid present in plaques in AD [46,52]. In particular, insulin resistance and T2D are major risk factors for the development of AD. Another line of similarities between AD and MD has been attributed to low-grade chronic inflammation in these conditions. Subclinical inflammation in the adipose tissue might provide an inflammatory stimulus towards central inflammatory regulation leading to neurodegeneration [53]. As several efforts to find effective therapies for AD have failed in the previous years, an alternate therapeutic strategy involving metabolic disease genes could be investigated. Recent studies have proposed repurposing T2D drugs for Alzheimer’s [54,55].

The pleiotropic nature of the 16 significant genes can be seen to be reflected in the analysis of their differential expression results. All of these genes show involvement in 3 types of disorders: ones related to CVD, immune system affecting disorders, and disorders related to the nervous system. For example, the gene TFE3 is implicated in HIV encephalitis. HIV is caused by an infection and is jointly classified under Infections and Immune System Diseases in MeSH. HIV encephalitis is the cognitive impairment due to HIV—a neurocognitive disorder. This gene is also implicated in polycystic ovary syndrome (PCOS), which is characterized, among others, by insulin resistance [56]. Thus, these genes are interesting candidates for investigating their causal link to such commonalities in different disease conditions, and more specifically, their role in MD co-morbidities, in the hope that some of them may be effective drug targets. 

The main advantage is that this method incorporates a single data-type. While several methods may include data from different sources, data integration can be a challenge and is generally a labor-intensive, error-prone task. Several methods require tuning of parameters or user inputs for the application of their algorithms. The proposed method is parameter-free, independent of the user input, and can be used as a first approach towards gene-prioritization. Its sole dependence on PPI data also enlarges its scope of application to other complex diseases for which quantitative data might not be available. Non-parametric significance testing allows for greater flexibility in the scope of distributions of centralities across different kinds of topologies. Hence, this method is robust to changes in underlying PPINs, and offers a solid background correction to avoid false positives. 

By definition of BC, single-degree genes have a centrality of 0. Although some MD genes may be single-degree genes, this method would be unable to identify those. Some low-degree genes, which may be connected in the MD network, may end up being at the edges of a random network, and hence cannot be assigned a centrality value. As Erten et al. [21] also observed, this type of method does penalize the highly connected genes. However, it is not designed to highlight all the MD related genes, only the genes with crucial topologies.

A key component of this analysis is the topology dependence of BC. Hence, results are best interpreted in the light of the topology of the starting PPIN. By using two different PPINs, we aim to correct for PPIN-specific artifacts stemming from the methods that were used to construct them. From the low overlap of interactions between HPRD and BioGRID, despite having a high overlap of the number of nodes, it is apparent that the two PPIs can be thought of as two independent networks. HPRD data were manually curated from literature, with most of the interactions included being backed by experimental systems, such as yeast two-hybrid methods. However, it was last updated in 2010. The BioGRID MV dataset contains interactions that have been validated by multiple resources. While the database is updated every month, the experimental pieces of evidence are considered to cover a much wider range, such as affinity capture, co-localization, co-purification, etc. It is likely that some interactions may occur under experimental conditions, but not in vivo. As more data gets added to these databases, the network structure is likely to change. In such a case, common candidates from different network topologies are likely to be the genes that remain central to the core network of interactions. BC is highly sensitive to the network structure, which is evident from the results. Hence, an overlap of the results from several different databases (and thus different networks) will increase the confidence level of the predictions. On the analysis of the MD network presented here, the major limitation stems from the limited overlap between datasets. Since not all of the known MD genes are present in the PPIN, the analysis is based on incomplete MD network reconstruction. As more reliable data become available, these findings can be reviewed in the light of the new information. However, the novel candidates identified in the study are strong candidates for expansion of the known MD genes network, based on the pathway and differential gene expression analysis results, and should be investigated further for their roles in the pathogenesis of MD and its co-morbidities.

## 4. Methods

For identification of genes with high relative BC, the pipeline consisted of:

(1) Data curation (a) Extraction of metabolic diseases from the MESH database, (b) Extraction of MD genes from the Comparative Toxicogenomics Database [30] (CTD), (c) PPI data curation, (d) HGNC [57] conversion of symbols.
(2) Reconstruction and analysis of the protein interaction network for MD genes.
(3) Construction and analysis of random networks for significance testing (Figure 1).

Data curation: The MeSH database (Medical Subject Headings 2019, accessed in October 2019) was used to retrieve the MeSH IDs for four categories of MD. Curated lists of disease-associated genes for four MeSH IDs were obtained from CTD for liver diseases (2069), metabolic diseases (1576), over-nutrition (220), and malnutrition (57). The non-redundant gene-list contained 3229 MD genes. These four categories include disease genes involved in metabolism. The complete table of MeSH IDs is available as Supplementary data (Supplementary Table S5). This list constituted our ‘seed’ MD genes. Furthermore, two PPI datasets were used: The Human Protein Reference Database [31] (HPRD, version 9, 2010) and BioGRID [32] multi-validated data set (Release 3.5.178, downloaded November 2019). HPRD and the multi-validated BioGRID database contain interactions based on experimental evidence such as yeast two-hybrid data, or multiple sources for validation. After removing incomplete entries, non-human interactions, and conversion of the gene names to the HGNC symbols, the largest component of each PPIN was retrieved and used for downside analyses. 

Reconstruction and analysis of the protein interaction network for MD genes: For each PPIN, disease-specific networks were extracted by including interactions between MD genes and their first neighbor. Interactions between first neighbors, if present, were also included. The giant component of this MD-specific network was used for further analysis (99% and 65% for HPRD and BioGRID, respectively). Single-degree nodes were removed unless they were a part of the seed gene list. Using the resulting topologies (‘MD networks’), the BC of each node was then computed using the parallelized version of the algorithm, available on the Networkx (https://networkx.github.io/ (accessed on 15 January 2021), version 2.3) platform (Python 3.7).

Construction and analysis of random networks for significance testing: To assess the significance of the centrality values in the MD network, they were compared with the values obtained by repeating the analysis and replacing the seed gene list with a set of randomly selected genes. For the comparisons to be fair, we used the same number of genes and degree-stratified sampling to obtain background networks of the same sizes and densities as the MD networks. For degree stratification, the code was designed to stratify the PPI networks such that each interval contained three degrees. The MD network was stratified and the number of genes in each interval was noted, and the same number of genes was chosen randomly from each corresponding interval for the entire PPI network. Because high-degree nodes are expected to display a higher BC, we sought to retrieve topologically influential nodes and correct for local effects by constructing random networks for comparison. We used the same number of genes and degree-stratified sampling to obtain background networks of the same sizes and densities as the MD networks. We then computed the BC for each node in 5000 such random networks. Under the assumption that no node has a particular topological effect in the MD network, the BCs for each node should be similar in MD-specific and random networks. Nodes with high connectivity will display a high BC whichever seed list is used to construct them, on average. In contrast, if a particular node is topologically interesting, for example by linking two subsystems that are relevant for MD, its BC might be higher in the MD network than is expected by chance, based on its degree. We calculated for each node the empirical *p*-value as:

(1) $$Pg=\frac{\left(rg+1\right)}{\left(ng+1\right)},$$

where *n_g_* is the total number of background networks that have been reconstructed where gene g is present, and *r_g_* is the number of times the BC of node g was greater in randomly constructed networks than in the MD network. (Supplementary File S1, Additional details on data analysis)

Multiple testing correction was applied using the Benjamini-Hochberg [58] method, and a list of significant genes (corrected *p*-value < 0.05) was obtained for each network. Overlap significance was calculated using the Hypergeometric test (Supplementary Table S5).

Pathway analysis: ConsensusPathDB [34] (CPDB) and Enrichr [33] were used to analyze the significant gene sets, highlighting significant pathways they were associated with. CPDB offers an over-representation tool that allows for a user-defined background gene list. All the nodes in the PPIN were used as background for CPDB. Enrichr offers results from several different pathway analysis databases, however, it generates its background data for comparison and significance testing. We also ran the pathway analysis and GO analysis for the 16 novel candidates, but no statistically significant results were found.

Differential expression analysis of novel significant genes: The online tool Harmonizome [35] was used to examine differential expression of the 16 novel genes found to be significant from the two PPIN. It uses transcriptomic (microarray) data from 233 Gene Expression Omnibus (GEO) datasets to identify disease-associated gene expression patterns. Strength of differential expression was the standardized score (Abs (standardized score)) = −log10 (*p*-value).

GWAS analysis was carried out for HPRD, however, for BioGRID, no results were obtained, perhaps, because the number of significant genes was lower. Results of pathway analysis using several resources such as Panther, Reactome, etc. were also provided by Enrichr. The complete set of results is available in the supplementary material (Supplementary Table S9). 

Software and computational processing: All the data processing and analysis pipelines were scripted in Python. Data visualization for graphs was done in Gephi. All the scripts for this pipeline are available on GitHub (https://github.com/sysbiolux/MD_network_map (accessed on 15 January 2021)).

## 5. Conclusions

Literature bias affords very high degrees to some genes while leaving several others understudied. These high-degree genes dominate analyses of networks created using literature curated databases. To highlight some of the topologically important nodes that may be central to a disease condition, we propose a simple, parameter-free method to obtain background-corrected BC scores. The method is applied to MD networks constructed using HPRD and BioGRID, and out of the top-scoring nodes in both the networks, 16 overlapping novel candidates were identified that are likely to contribute to the development and/or progression of MD and its co-morbidities. These candidates need to be further investigated to ascertain their role in MD. This is important from the perspective of developing effective therapies for MD and associated co-morbidities.

## Figures and Tables

**Table 1:** Novel candidates identified in the study; list of 16 novel genes found to be significant in both HPRD and BioGRID PPINs.

Symbol	Name
ALOX5	arachidonate 5-lipoxygenase
BATF	basic leucine zipper transcription factor, ATF-like
BNIPL	BCL2/adenovirus E1B 19kD interacting protein-like
DUSP22	dual specificity phosphatase 22
FBLN5	fibulin 5
GPC1	glypican 1
IL5RA	interleukin 5 receptor, alpha
OPRK1	opioid receptor, kappa 1
PLSCR3	phospholipid scramblase 3
PMPCB	peptidase (mitochondrial processing) beta
PTPN11	protein tyrosine phosphatase, non-receptor type 11
RNF128	ring finger protein 128, E3 ubiquitin protein ligase
S100A7	S100 calcium-binding protein A7
SNCG	synuclein, gamma (breast cancer-specific protein 1)
STIM2	stromal interaction molecule 2
TFE3	transcription factor binding to IGHM enhancer 3

**Table 2:** Differential gene expression analysis; some of the significant genes and their differential expression in different conditions based on Gene Expression Omnibus (GEO) datasets. The complete table is available in supplementary files.

Gene	Upregulation	Downregulation
ALOX5	Senescence, Rotavirus infection of children, Down Syndrome, Neurological pain disorder, Severe combined immunodeficiency (SCID)	Macular degeneration, Human immunodeficiency virus infection (HIV), Atherosclerosis
BATF	Glaucoma, Human immunodeficiency virus infection (HIV), Appendicitis, Oligodendroglioma, Multiple Sclerosis (MS), Severe acute respiratory syndrome (SARS), Diabetic Nephropathy	Chronic obstructive pulmonary disease (COPD), Cardiac Hypertrophy, Scleroderma, Retinoschisis
IL5RA	Cardiac Failure, Pauciarticular juvenile arthritis	Down Syndrome, Lung Injury, Familial combined hyperlipidaemia
PLSCR3	Erectile dysfunction, Breast Cancer, Bipolar Disorder, Appendicitis, Papillary Carcinoma of the Thyroid, Bipolar Disorder	Atherosclerosis, Cardiomyopathy, Myocardial Infarction
S100A7	Type 2 diabetes mellitus, Post-traumatic stress disorder (PTSD), Eczema	-

## Data Availability

The code used for this analysis is available on GitHub: https://github.com/sysbiolux/MD_network_map.

## Supplementary Materials

The following are available online at https://www.mdpi.com/2079-7737/10/2/107/s1, Figure S1: Network view of one of the novel candidates identified, ALOX5, in HPRD. ALOX5 is highly central to some of the known MD genes—ACTB, ALOX5AP, and COTL1, and forms an important link between dense clusters of MD gene COTL1, and other non-MD first neighbors, Figure S2: Network view of one of the novel candidates identified, ALOX5, in BioGRID. Similar to its network in HPRD, ALOX5 here is connected to a single degree, but known MD genes ALOX5AP, COTL1, and LTC4S. It also links to clusters of non-MD first neighbors DICER1 and GRB2. Notice that ALOX5 by itself has a low degree, but is a crucial link to the single degree, known MD genes in the network. Thus, ALOX5 becomes a topologically important node, Table S1: List of Seed genes (Initial and Processed), Table S2: Common genes among Top 1000 genes (based on *p* values) from HPRD and Bi-oGRID, Table S3: List of Significant genes in HPRD, Table S4: List of Significant genes in BioGRID, Table S5: Miscellaneous data, Table S6: List of common pathways, Table S7: Pathway analysis for significant genes in HPRD, Table S8: Pathway analysis for significant genes in BioGRID, Table S9: Differential Gene Expression analysis for 16 common novel significant genes.

## Abbreviations

AD	Alzheimer’s disease
CTD	Comparative Toxicogenomics Database
BC	Betweenness centrality
CVD	Cardiovascular diseases
HGNC	HUGO Gene Nomenclature Committee
HPRD	Human Protein Reference Database
MD	Metabolic diseases
PPIN	Protein-protein interaction network
T2D	Type 2 diabetes
