# Supplementary material for: Degree Adjusted Large-Scale Network Analysis Reveals Novel Putative Metabolic Disease Genes
Source: Biology (Basel). 2021 Feb 3;10(2):107. doi: 10.3390/biology10020107 (PMC7913176; doi:10.3390/biology10020107)
Supplement: Supplementary file 1 [file biology-10-00107-s001.zip › Revised_supplementary/Supplementary_File_1.docx]

**Supplementary Data File 1**

1. **Supplementary Methods:**

Details of the random network construction are as follows:

1. The degree distribution of the MD network for each of the PPI was determined.
2. Degree distribution of the PPI network was determined, and the network was stratified into different groups with a minimum difference of 3 between each of the groups.
3. Equivalent number of genes were sampled from each group as the number of genes in the corresponding group in the MD network.

Thus, each of the random networks was constructed using the same number of nodes and was designed to be topologically similar to the original MD network in that PPI network.

Data analysis and significance testing:

For a series of 5000 random runs, a data selection strategy was adopted which was as follows:

1. For a gene present in the original MD list, its betweenness centrality value for the random network was selected if the gene was also present in the random gene list for the network. Otherwise, the value was discarded.
2. For a gene absent from the original MD list, its betweenness centrality value for the random network was selected when it was absent from the random gene list. If the gene was present in the random list, its corresponding centrality value was discarded.

This procedure was adopted because if a gene is present as an MD node, all of its first neighbors are selected. In case of highly connected genes such as TP53, EGFR, etc., presence or absence in the gene-list leads to either a very high value of centrality, or a very low value of the centrality, i.e., a bimodal distribution. Thus, on calculating the average in such a case, the centrality of a node in the random network would not be reflective of the true values of the node in reference networks. Comparison between the MD network centrality vs its random network centrality would lead to artificially increasing the node’s p-values.

**2. Supplementary Figures:**


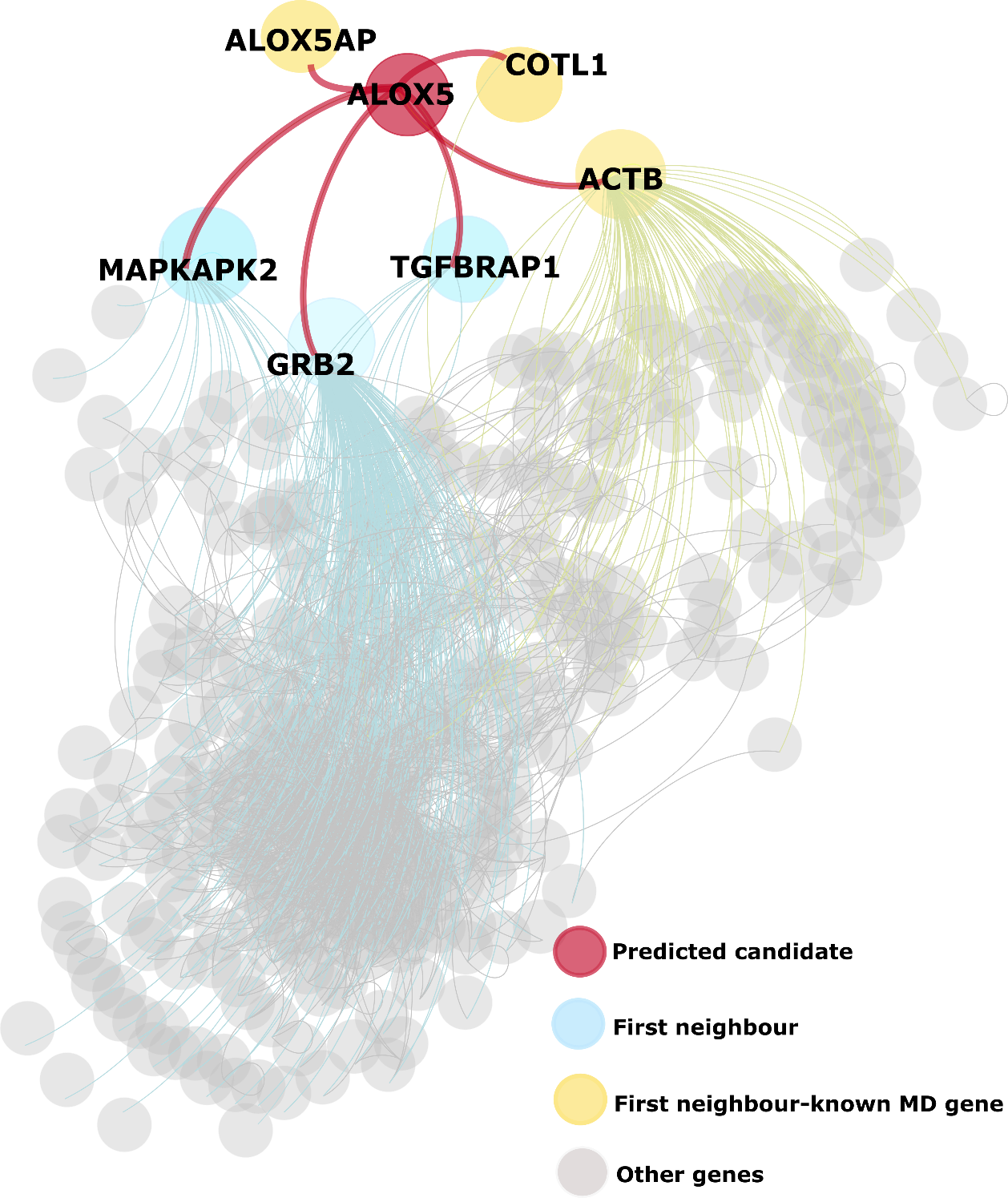


Fig. S1: Network view of one of the novel candidates identified, ALOX5, in HPRD. ALOX5 is highly central to some of the known MD genes – ACTB, ALOX5AP, and COTL1, and forms an important link between dense clusters of MD gene COTL1, and other non-MD first neighbors.


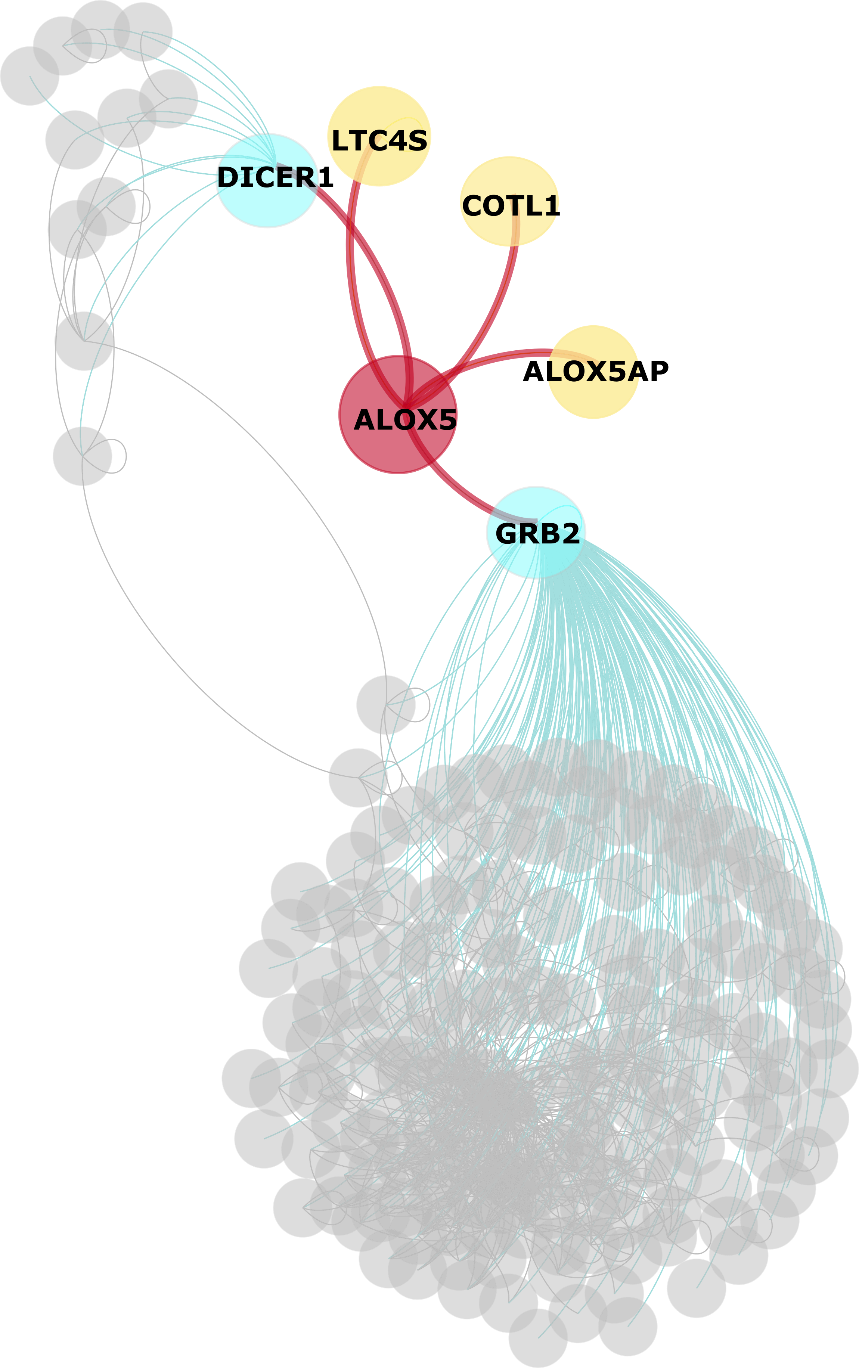


Fig. S2: Network view of one of the novel candidates identified, ALOX5, in BioGRID. Similar to its network in HPRD, ALOX5 here is connected to single degree, but known MD genes ALOX5AP, COTL1, and LTC4S. It also links to clusters of non-MD first neighbors DICER1 and GRB2. Notice that ALOX5 by itself has a low degree, but is a crucial link to the single degree, known MD genes in the network. Thus, ALOX5 becomes a topologically important node.
